# Hypoxic-induced truncation of voltage-dependent anion channel 1 is mediated by both asparagine endopeptidase and calpain 1 activities

**DOI:** 10.18632/oncotarget.24377

**Published:** 2018-01-31

**Authors:** Hadas Pahima, Simona Reina, Noa Tadmor, Daniella Dadon-Klein, Anna Shteinfer-Kuzmine, Nathalie M. Mazure, Vito De Pinto, Varda Shoshan-Barmatz

**Affiliations:** ^1^ Department of Life Sciences and the National Institute for Biotechnology in the Negev, Ben-Gurion University of the Negev, Beer-Sheva 84105, Israel; ^2^ Department of Biomedicine and Biotechnology, University of Catania and National Institute for Biomembranes and Biosystems, Section of Catania, Catania 95125, Italy; ^3^ Department of Biological, Geological and Environmental Sciences, University of Catania, Catania 95125, Italy; ^4^ Institute for Research on Cancer and Aging of Nice, University of Nice Sophia-Antipolis, Centre Antoine Lacassagne, Nice 06189, France; ^5^ Present address: INSERM U1065, C3M, Nice 06204, France

**Keywords:** asparagine endopeptidase, calpain, hypoxia, VDAC1

## Abstract

The voltage-dependent anion channel 1 (VDAC1), an outer mitochondria membrane (OMM) protein, serves as a mitochondrial gatekeeper, mediating the transport of nucleotides, Ca^2+^ and other metabolites across the OMM. VDAC1 also plays a central role in mitochondria-mediated apoptosis by facilitating the release of apoptotic proteins and by association with both pro- and anti-apoptotic proteins. Tumor cells, which are constantly exposed to hypoxic conditions, affect the cell via the transcription factor hypoxia-inducible factor (HIF) that induces transcriptional activity. In cultured cells and in lung cancer patients, hypoxia induces VDAC1 truncation at the C-terminus (VDAC1-ΔC). However, the molecular mechanisms involved in VDAC1-ΔC formation are unknown. Here, we show that hypoxia-induced VDAC1-ΔC formation is inhibited by the Ca^2+^ chelator BAPTA-AM, by calpain inhibitor-1, by inhibitor of the asparagine endopeptidase (AEP) and by si-RNA targeting HIF1-α or Ca^2+^-activated protease calpain-1 expression but not that of calpain-2. Finally, VDAC1-ΔC expressed in bacteria and reconstituted into a planar lipid bilayer exhibited decreased channel conductance relative to the full-length protein, yet retained voltage-dependent conductance. These findings suggest that hypoxia, acting via HIF-1α expression, leads to VDAC1 cleavage involving the activation of calpain 1 and AEP.

## INTRODUCTION

Defective mitochondrial function is associated with hypoxia, a situation when insufficient amounts of oxygen reach a given tissue [1]. Prolonged and severe hypoxia can be toxic to normal cells and has been invoked in many pathophysiological conditions, such as type 2 diabetes [2], Alzheimer's disease [3], cardiac ischemia/reperfusion injury [4] and cancer [5–7]. However, cancer cells undergo genetic and adaptive changes that allow them to survive and even proliferate in hypoxic environments [8].

Several hypoxic signaling pathways have been shown to influence tolerance to hypoxia and other phenotypes relevant to cancer. These include the activation of hypoxia-inducible transcription factors (HIF1, HIF2, HIF3), which induce the expression of a large number of genes involved in glycolysis, angiogenesis, pH regulation, and cell motility [8–12]. In addition, hypoxia induces pro-survival changes in the expression of genes that suppress apoptosis [12, 13] and support autophagy [10, 12]. Hypoxia also enhances receptor tyrosine kinase-mediated signaling [12, 14], thus suppressing immune reactivity [12, 15] and metastasis [12, 16]. In addition, hypoxia contributes to a loss of genomic stability through the increased generation of reactive oxygen species (ROS) [12, 17] and the down-regulation of DNA repair pathways [12, 18]. In, addition, tumors exposed to a limiting oxygen microenvironment acquire resistance to chemotherapy-induced apoptosis [12, 19, 20] and present reduced responses to radiotherapy [8].

Recently, hypoxic conditions were shown to cause hyper-fusion of mitochondria, leading to the formation of enlarged organelles, and to induce cleavage at the C-terminal end of the voltage-dependent anion channel 1 (VDAC1) to form a 26 kDa protein band (VDAC1-ΔC). Such cleavage was prevented upon silencing HIF-1α expression [21, 22]. VDAC1-ΔC was detected in tumor tissues of non-small cell lung carcinoma patients, being found more frequently in large and late-stage tumors [21, 22]. It was proposed that hypoxia, by inducing formation of VDAC1-ΔC, confers selective protection from apoptosis that allows maintenance of ATP and cell survival in hypoxia [19]. VDAC1-ΔC formation was, moreover, found to be dependent on p53 or p73, and on local microfusion between mitochondria and endolysosomes observed upon hypoxia. It was also suggested that asparagine endopeptidase (AEP) mediates VDAC1 truncation [21, 22].

Located in the outer mitochondrial membrane (OMM), VDAC1 assumes a crucial position in the cell, mediating the transfer of metabolites, nucleotides, Ca^2+^ and other ions, thus controlling metabolic cross-talk between mitochondria and the rest of the cell [23]. Moreover, VDAC1 is a key player in mitochondria-mediated apoptosis, participating in the release of mitochondrial pro-apoptotic proteins to the cytosol [e.g. cytochrome *c*, AIF, *SMAC*/*Diablo*] [24, 25], and interacting with apoptosis regulatory proteins, such as members of the Bcl-2 family of proteins [26–28] and hexokinase [26, 29]. Thus, VDAC1 is involved in controlling the fate of the cancer cells [23, 26, 30].

It has also been shown that VDAC1 can be cleaved by partially purified mitochondrial m-calpain to yield truncated VDAC1 in a Ca^2+^-dependent manner, with such cleavage being inhibited by the non-competitive calpain inhibitor PD150606 [31]. In addition, this cleavage induces conformational changes in VDAC1 and in Bax binding to VDAC1. Still, only limited research on the physiological functions of VDAC1 cleavage by mitochondrial calpain has appeared. Calpains are a 15-member family of Ca^2+^-activated cysteine proteases localized to the cytosol and mitochondria, several of which have been shown to regulate apoptosis and necrosis [32]. Calpain-1 [μ-calpain] requires micromolar levels of Ca^2+^, while calpain-2 [m-calpain] requires millimolar levels of Ca^2+^. Both μ-calpain [33, 34] and m-calpain [31, 34] are located in the mitochondrial inter-membrane space [IMS]. Calpain-10 is found in all mitochondrial compartments, with the majority of activity being detected in the matrix [35]. The μ- and m-calpain heterodimers regulate and are even required for numerous physiological processes ranging from embryogenesis to cell adhesion [36]. Calpains are, however, also involved in a large number of pathological conditions, such as diabetes and Alzheimer's disease [37].

The pro-apoptotic activity of calpains includes cleavage of caspase 3 and/or AIF [32]. Calpain has, moreover, been shown to participate in the destruction of HIF-1α, transiently expressed under hypoxic conditions [38], implying that calpain-evoked destruction of HIF-1α must be considered as an alternative system to the established proteasomal degradation pathway known to affect HIF-1α levels [38].

The data presented above led us to examine the possibility that calpain proteases also participate in VDAC1 cleavage under hypoxic conditions. As such, we addressed VDAC1 cleavage induced upon hypoxia and now report the requirement of Ca^2+^ and calpain activity for hypoxia-induced truncation of a VDAC1 C-terminal domain. In addition, electrophysiological characterization of recombinant truncated forms of VDAC1 was performed.

## RESULTS

In this study, we used both CoCl_2_ and an O_2_ level-controlled incubator to characterize VDAC1 truncation as induced upon hypoxic conditions. CoCl_2_ has been widely used as a hypoxic mimetic in cell culture [39] and was shown to act by stabilizing HIF-1α [40]. CoCl_2_ also enhanced the transcription of a set of hypoxia-responsive genes, including genes encoding the Glut-1, glucose transporter and several enzymes of glycolysis [41, 42].

**Figure 1:**
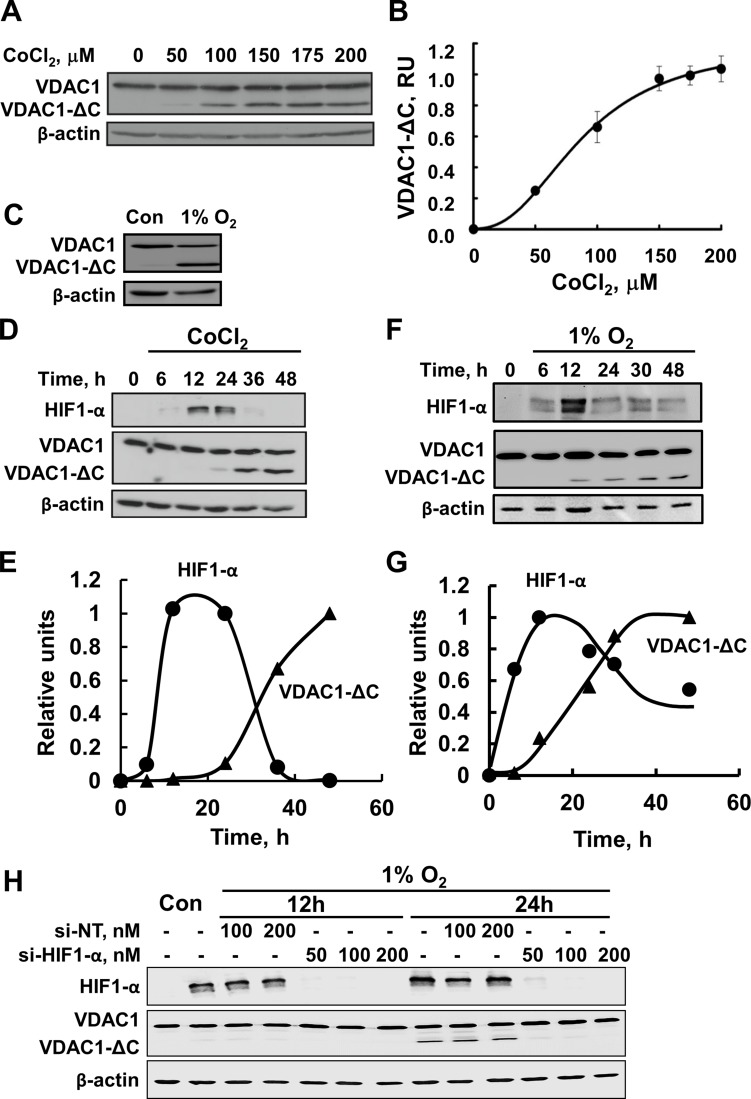
Hypoxia, as induced by CoCl_2_ or low oxygen, leads to VDAC1 truncation (**A**) HeLa cells were incubated overnight in a serum-free DMEM, followed by addition of different concentrations of CoCl_2_ for 24 h. Samples were subjected to SDS-PAGE, followed by immunoblotting using antibodies against the N-terminal domain of VDAC1. (**B**) Quantitative analysis of the results of three independent experiments carried out using multi-gauge software and presented in relative units. (**C**). HeLa cells were exposed to 1% O_2_ for 48 h and then analyzed by immunoblotting. (**D**) HeLa cells were incubated for 16 h in a serum-free DMEM, followed by addition of 150 μM CoCl_2_ for various times, and analyzed by immunoblotting, using either anti-HIF1-α or anti-VDAC1 antibodies. (**E**) HIF1-α and VDAC1-ΔC levels were analyzed and are presented as relative units. (**F**) HeLa cells were exposed to 1% O_2_ for the indicated time and then analyzed by immunoblotting using either anti-HIF1-α or anti-VDAC1 antibodies. (**G**) Quantification of VDAC1-ΔC and HIF1-α levels is presented, relative to the maximal level of each protein, defined as 1.0, and normalized to β-actin levels. A representative of three similar experiments is shown. (**H**) HeLa cells were treated under hypoxic conditions (1% O_2_) for 12 or 24 h with 50, 100 or 200 nM of si-RNA against HIF1-α and analyzed for VDAC1, VDAC1-ΔC, and HIF1-α levels by immunoblotting using specific antibodies.

Previous studies demonstrating that upon hypoxia (1% O_2_) VDAC1 was cleaved, as reflected in the appearance of a 26 kDa protein band [19]. To further characterize the truncation process and the channel properties of the truncated VDAC1 obtained in response to hypoxia produced either by reducing O_2_ levels or in the presence of CoCl_2_. HeLa cells were incubated with different concentrations of CoCl_2_ (Figure 1A, 1B) or in 1% O_2_ (Figure 1C), leading to the formation of truncated VDAC1 with a molecular mass of about 26 kDa, as visualized by immunoblotting using antibodies against the N-terminal region of VDAC1. Quantitative analysis of the levels of truncated VDAC1 protein formed as a function of CoCl_2_ concentration showed a concentration-dependent increase, with 50% of the maximal level being obtained at 75 μM CoCl_2_, and the maximal level appearing in the presence of about 175 μM CoCl_2_ (Figure 1B). As reported previously [19], VDAC1 was truncated at the C-terminus (VDAC1-ΔC), since only antibodies against the N-terminal domain recognized the truncated protein.

To demonstrate that CoCl_2_-mimicked hypoxia-induced VDAC1 truncation involves HIF-1α, the relationship between the appearances of HIF-1α and VDAC1-ΔC was examined (Figure 1D, 1E). Transient expression of HIF-1α was observed, with the highest level being obtained after 12 h of exposure to CoCl_2_. This level was slightly decreased after 24 h, and the protein completely disappeared after 36 h. Interestingly, the formation of VDAC1-ΔC follows the appearance of HIF-1α (Figure 1E). Similar results were obtained when hypoxia was induced by 1% O_2_ (Figure 1F, 1G). It should be noted, however, that the expression of HIF-1a was always transient, with a maximal level being observed between 12 and 24 h. This timing, dependent on cell density, the number of cell passages and the source of the cells, as well as how fast the cells were denaturated following their transfer from conditions of hypoxia to normoxia, an event that results in HIF-1a degradation.

Finally, under hypoxic conditions, siRNA specific to HIF-1α decreased HIF-1α expression by over 90% and completely inhibited the formation of VDAC1-ΔC (Figure 1H).

These results confirm that induction of HIF-1α expression by CoCl_2_ or 1% O_2_ leads to VDAC1 cleavage to form VDAC1-ΔC [19].

### Hypoxia-induced VDAC1 truncation involves Ca^2+^ and calpain

**Figure 2:**
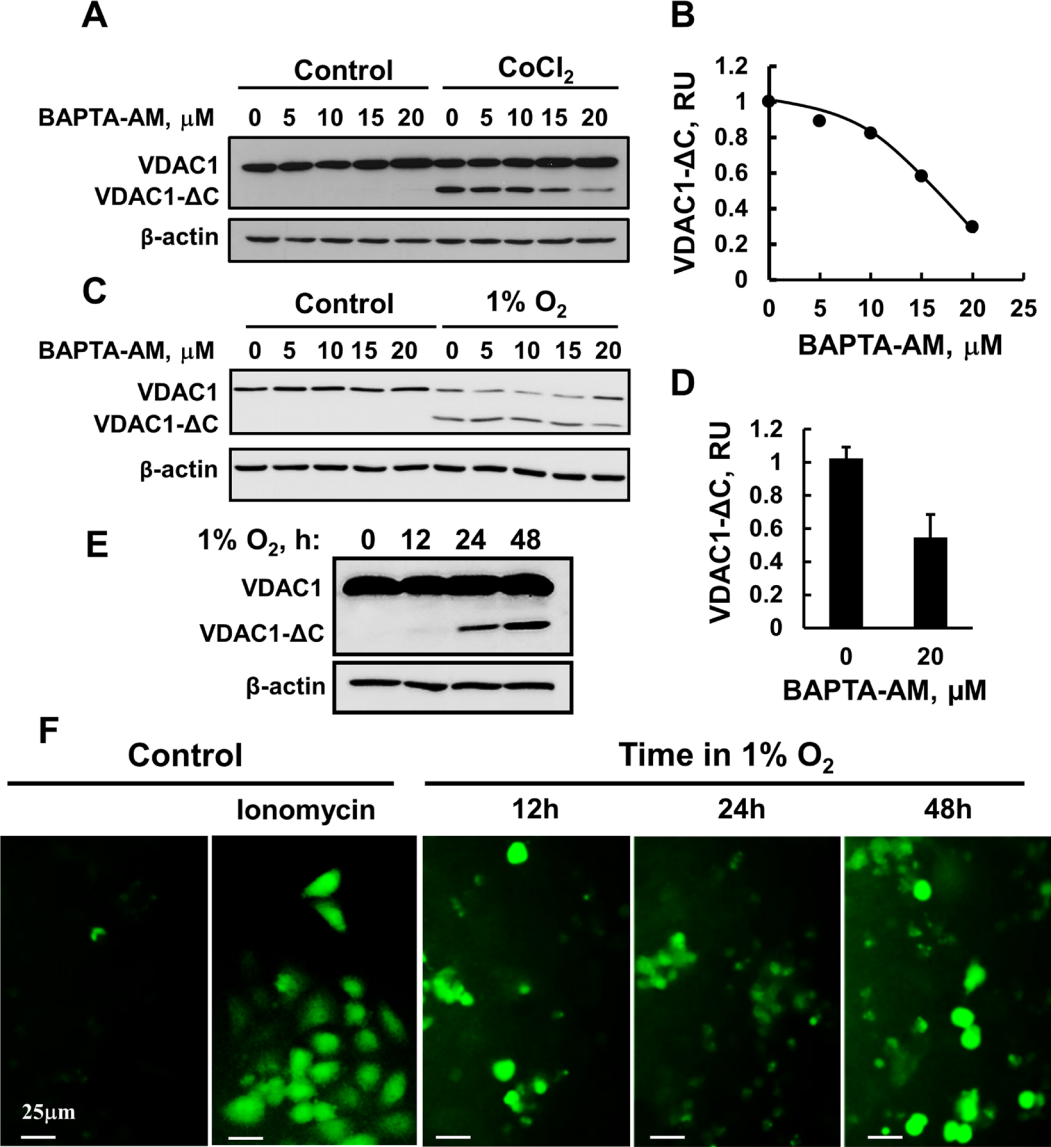
BAPTA-AM inhibits VDAC1-ΔC formation (**A**) HeLa cells were incubated for 24 h in a serum-free DMEM with 150 μM CoCl_2_ and the indicated concentrations of BAPTA-AM, and the formation of VDAC1-ΔC was assessed by immunoblotting. A representative experiment is presented. (**B**) The results were quantified relative to β-actin levels and are presented in relative units (RU). (**C**) HeLa cell were exposed to 1% O_2_ for 48 h in the absence or presence of the indicated concentration of BAPTA-AM, immunoblotted and analyzed for VDAC1-ΔC levels (**D**). (**E**) HeLa cells were transfected to express the Ca^2+^ indicator GCaMP5 as described in Experimental Procedures and 24 h post-transfection, were grown in either normoxic or hypoxic conditions. Cells exposed to normoxic or hypoxic conditions were analyzed for the 12, 24 and 48 h later, formation of VDAC1-ΔC using anti-VDAC1 antibodies (**E**) or fluorescent cells were visualized using a fluorescence microscope (**F**). Where indicated, cells in normoxic conditions were incubated for 15 min with ionomycin (5 μM) and then visualized.

Although the endolysosomal asparagine endopeptidase (AEP) was proposed to mediate VDAC1 truncation [21, 22], we asked whether Ca^2+^-dependent proteases are involved in VDAC1 truncation. The intracellular Ca^2+^ concentration ([Ca^2+^]i) was reduced using the Ca^2+^chelator BAPTA-AM (Figure 2A–2D). HeLa cells were incubated with CoCl_2_ in the absence or presence of different concentrations of BAPTA-AM for 24 h. Under these conditions, BAPTA-AM decreased VDAC1-ΔC appearance (Figure 2A, 2B), suggesting that Ca^2+^ is required for VDAC1 truncation. Treatment of control cells with the same concentration of BAPTA-AM had no effect on VDAC1 levels. Similar results were obtained when hypoxia was induced using 1% O_2_ (Figure 2C, 2D).

Next, we analyzed the effect of hypoxia on [Ca^2+^]i levels using the genetically encoded Ca^2+^ indicator GCaMP5 [43]. VDAC1-ΔC was formed in GCaMP5 expressing cells, following exposure to hypoxic conditions for 12, 24 and 48 h (Figure 2E). Fluorescent images of HeLa cells expressing GCaMP5 were acquired immediately after removing the culture from the incubator set for hypoxic conditions. GCaMP5-expressing cells showed fluorescence only if grown under hypoxia or in response to ionomycin (Figure 2F). Both the fluorescence intensity and the number of fluorescent cells were increased with the time of exposure to hypoxic conditions (Figure 2F).

**Figure 3:**
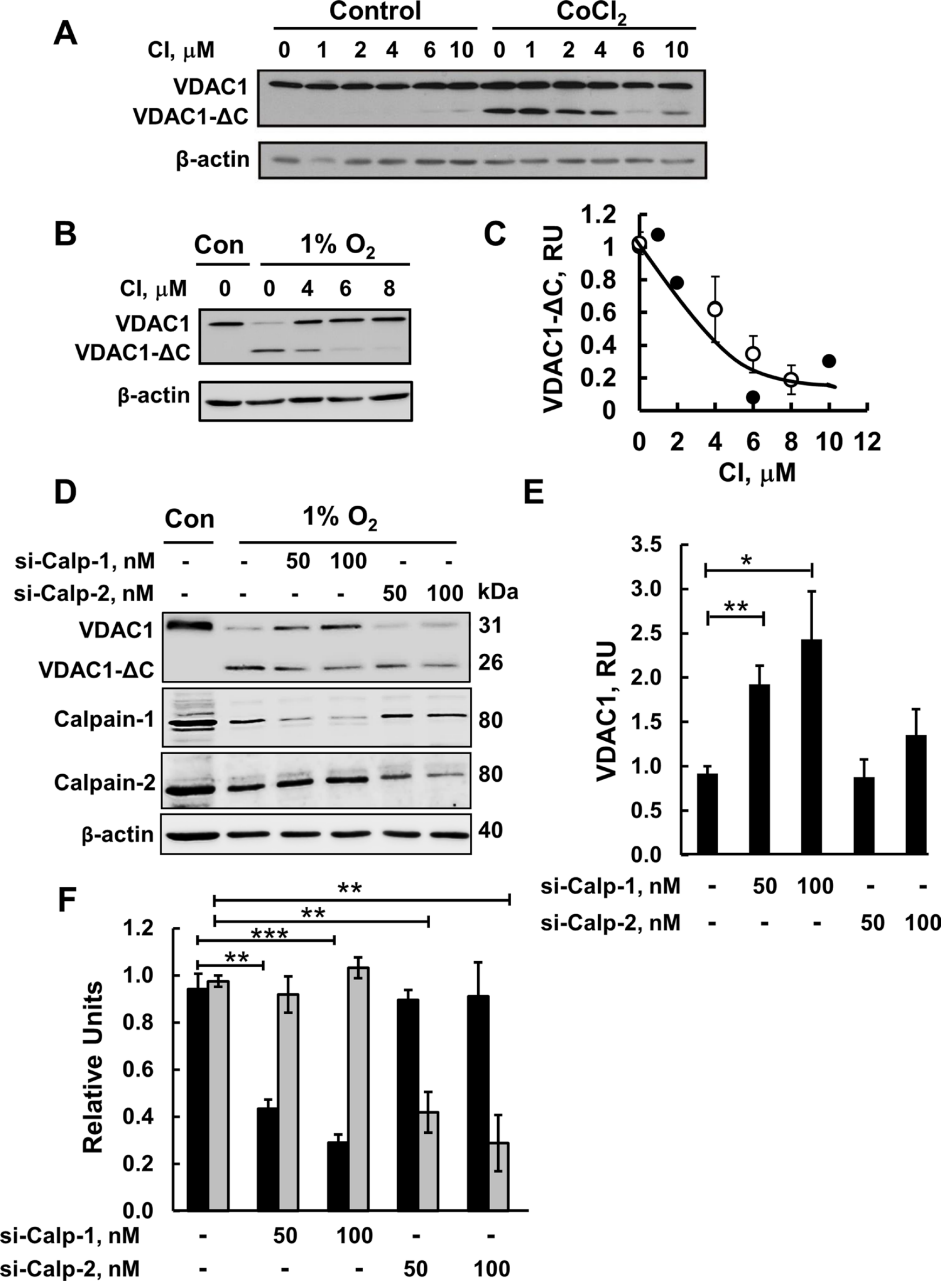
Calpain inhibitor I and siRNA against calpain 1 inhibit VDAC1-ΔC formation (**A**) HeLa cells were incubated for 24 h in serum-free medium with or without 150 μM CoCl_2_ and in the absence or presence of the indicated concentration of calpain inhibitor I (CI). VDAC1-ΔC levels were analyzed by immunoblotting (see C). (**B**) Immunoblotting of cells incubated in 1% O_2_ in the absence or presence of CI. (**C**) Quantification of VDAC1-ΔC levels from (A, full circles) and (B, empty circles) is presented, relative to β-actin levels. (**D**) HeLa cells were treated under hypoxic conditions (1% O_2_) for 48 h with 50 or 100 nM of si-RNA against calpain-1 (si-Calp-1) or calpain-2 (si-Calp-2) and analyzed for VDAC1, VDAC1(ΔC), calpain-1 and calpin-2 levels by immunoblotting using specific antibodies. (**E**) Quantitative analysis of VDAC1 levels from three similar experiments as in (D). (**F**) Quantitative analysis of calpain-1 (black bars) and calpain-2 (grey bars) levels. The results are the mean ± SE from five similar experiments as in (A). ^*^P ≤ .0.01, ^**^P ≤ .001; ^***^P ≤ .0001.

Since Ca^2+^ is a known activator of the calpain family of proteases, corresponding to a group of Ca^2+^-regulated neutral cysteine proteases [34], several members of which are present in mitochondria [32, 33, 44], we evaluated the involvement of calpains in the truncation of VDAC1. The effect of calpain inhibitor 1 on the formation of VDAC1-ΔC as induced by CoCl_2_ or 1% O_2_ was tested (Figure 3A–3C). Calpain inhibitor 1 (CI) inhibited the formation of VDAC1-ΔC, induced by either CoCl_2_ or 1% O_2,_ in a concentration-dependent manner, with an IC_50_ of 4 μM. Complete inhibition was obtained at about 10 μM. These results suggest that VDAC1 truncation can be executed by Ca^2+^-activated calpains.

Since calpain inhibitor 1 is not calpain isoform-specific, we used siRNA specific to either calpain-1 (si-hCalpain-1) or calpain-2 (si-hCalpain-2) on cells that perform hypoxia (1% O_2_)-induced VDAC1 truncation to identify the specific calpain that mediates VDAC1 cleavage (Figure 3D–3F). si-hCalpain-1 and si-hCalpain-2 (100 nM) decreased the expression levels of calpain-1 and calpain-2 by about 70% each (Figure 3D, 3F). The effects of si-hCalpain1 and si-hCalpain-2 on the formation of VDAC1-ΔC, as induced by hypoxia (1% O_2_), was analyzed in HeLa cells. While si-hCalpain-1 inhibited VDAC1 truncation, as reflected in the increase in full-length VDAC1 and decrease in VDAC1-ΔC levels, si-hCalpain2 treatment showed no significant effect on the formation of VDAC1-ΔC (Figure 3D and 3E). In normoxic conditions, si-hCalpain-1 and si-hCalpain-2 had no effect on VDAC1 expression levels (data not shown). Therefore, we concluded that calpain-1 is involved in VDAC1 truncation under hypoxic conditions.

### Asparagine endopeptidase inhibitor inhibits hypoxia-induced VDAC1-ΔC formation

**Figure 4:**
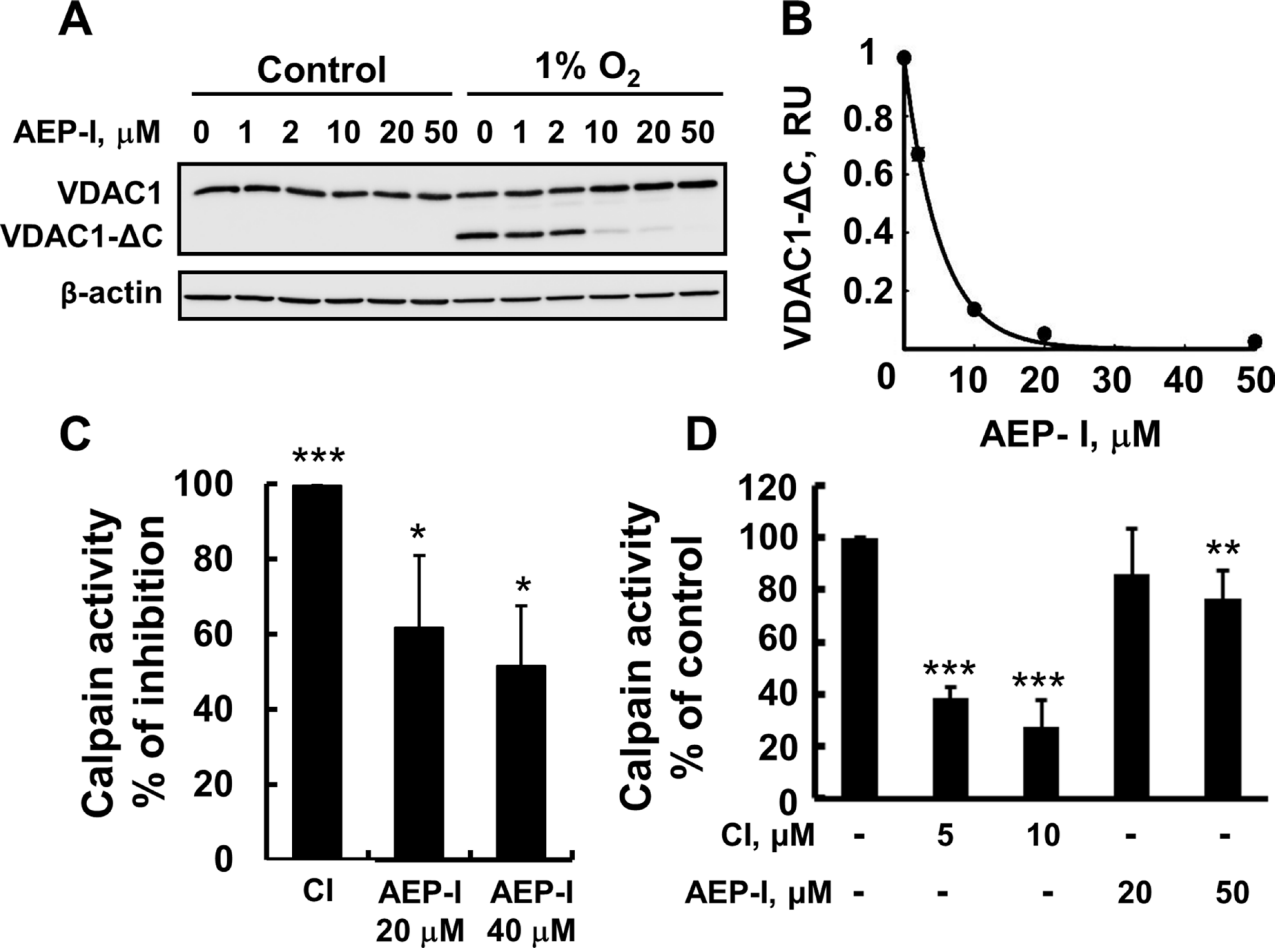
AEP inhibitor inhibits VDAC1-ΔC formation (**A**) HeLa cell exposed to 1% O_2_ for 48 h in the absence or presence of the indicated concentration of AEP inhibitor (AEP-I), immunoblotted and analyzed for VDAC1-ΔC levels. (**B**) Quantitative analysis of VDAC1 levels from three similar experiments as in (A). (**C**) Purified calpain-1 was incubated with 20 or 40 μM of AEP-I or 10 μM of CI for 5 min and calpain activity was analyzed as described in Experimental Procedures. (**D**) HeLa cell exposed to 1% O_2_ for 24 h in the absence or presence of the indicated concentration of AEP-I, and then assayed for calpain activity as described in Experimental Procedures. Results are the mean ± SEM (*n* = 3) ^*^P ≤.0.01, ^**^P ≤.001; ^***^P ≤.0001.

It has been proposed that hypoxia induces mitochondrial-endolysosomal crosstalk, leading to VDAC1 truncation by asparagine endopeptidase (AEP, or legumain), a cysteine endopeptidase associated with lysosomes [21, 22]. Therefore, we targeted AEP using the specific inhibitor MV026630 [45]. This AEP inhibitor (AEP-I) strongly reduced the formation of VDAC1-ΔC in a concentration-dependent manner, with half maximal inhibition (IC_50_) being obtained at 4 μM (Figure 4A, 4B).

As both AEP and calpain are cysteine proteases, we next tested whether AEP inhibitor also inhibited the activity of purified and/or cellular calpain (Figure 4C, 4D). At the concentration used (20 μM), AEP-I decreased the activity of purified calpain by about 60% (Figure 4C). The effect of AEP-I on calpain activity in cells exposed to normoxic and hypoxic (1% O_2_) conditions was also tested (Figure 4D). Calpain activity was slightly (10–20%) increased in cells exposed to hypoxic conditions (data not shown), in agreement with the reported hypoxia-inducing up-regulation of calpain activity and mRNA expression in pulmonary artery endothelial cells [46]. AEP-I decreased calpain activity in cells exposed to normoxia or hypoxia. For comparison, and as expected, the calpain-specific inhibitor strongly inhibited calpain activity (Figure 4C, 4D). Thus, the decrease in VDAC1-ΔC formation mediated by AEP inhibitor could be partially due to inhibition of calpain activity.

### Characterization of VDAC1-ΔC channel activity

Previously [19], we reported that VDAC1-ΔC purified from cells exposed to hypoxic conditions and reconstituted into a planar lipid bilayer possesses similar channel activity as full-length VDAC1. This is unexpected as the pore size of a channel composed of 15 or 16 β-stands instead of the native 19 β-strands should be smaller and, accordingly, present decreased channel conductance. This raises the possibility that upon cleavage of a peptide bond at the C-terminal of membrane embedded VDAC1, the protein remains intact as a β-barrel, also following protein purification. Separation of the C-terminal fragment from the larger VDAC1 segment occurs only upon SDS-PAGE. This may explain the similar conductance of purified VDAC1 and purified VDAC1-ΔC.

**Figure 5:**
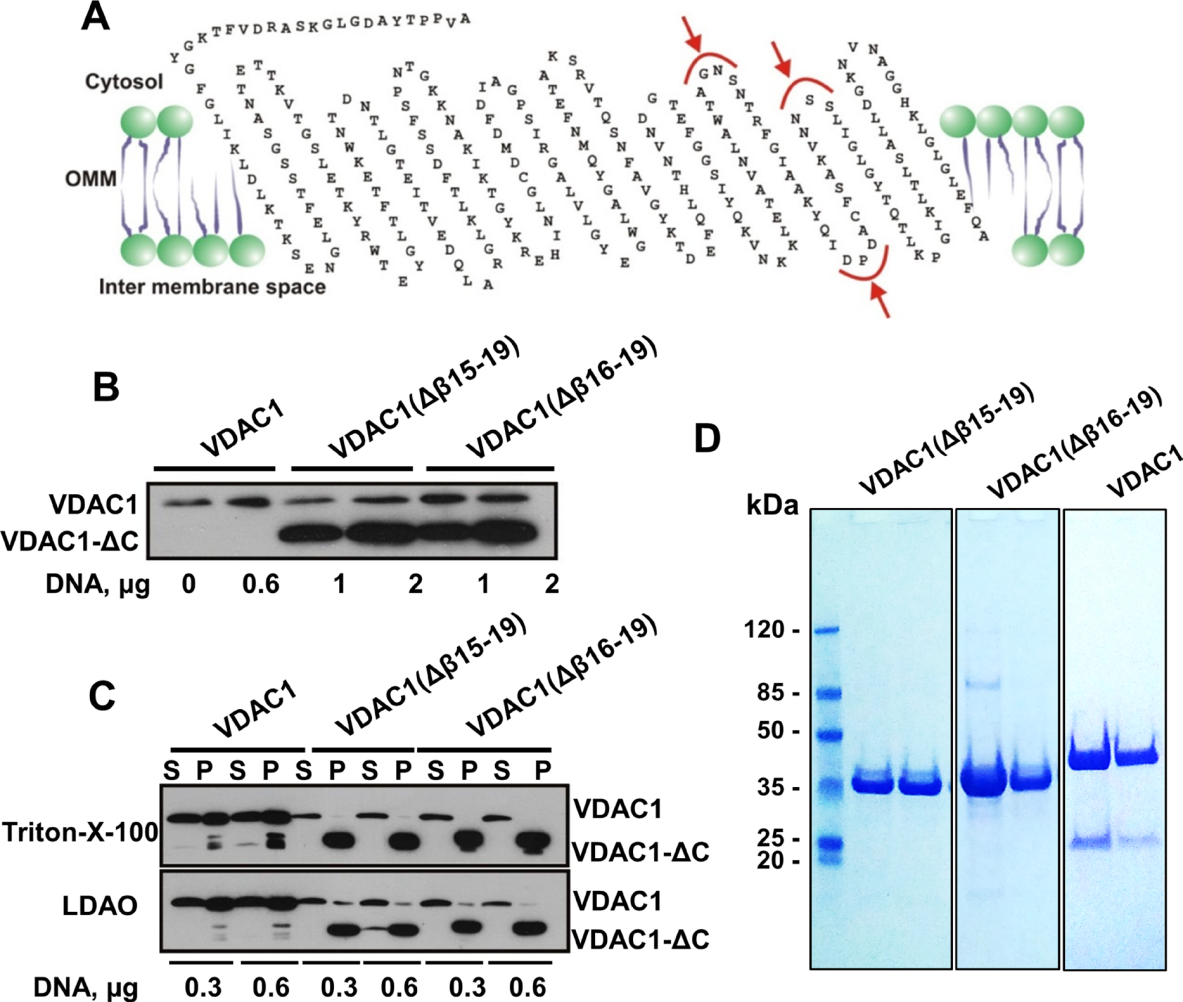
Expression of recombinant C-terminally truncated VDAC1 (**A**) VDAC1 membrane topology model presenting proposed calpain cleavage sites (red arrows). The two truncated VDAC1 versions produced were VDAC1(Δ229-283), with β-strands 15 to 19 deleted (VDAC1(Δβ15-19)), and VDAC1(Δ241-283), with β-strands 16 to 19 deleted (VDAC1(Δβ16-19)). (**B**) HEK-293 cells were transfected with plasmid pcDNA3.1 encoding VDAC1 (0.6 mg), VDAC1(Δβ15-19) or VDAC1(Δβ16-19) (1 or 2 mg) using calcium phosphate. Expression of the recombinant proteins was analyzed 24 h post-transfection by immunoblotting. (**C**) HEK-293 cells expressing truncated VDAC1 constructs were trypsinized 24 h post-transfection, washed with PBS, and proteins were solubilized with LDAO (2%) or Triton X-100 (3%) as described in Experimental Procedures. Supernatant (S) and pellet (P) obtained following centrifugation at 20,000g were analyzed for the presence of the different VDAC1 versions by immunoblotting. The pellet was dissolved in 5% of the original volume, thus it is about 20-fold more concentrated, relative to the supernatant. (**D**) Coomassie blue staining of recombinant VDAC1, VDAC1(Δβ15-19) and VDAC1(Δβ16-19), expressed in bacteria and purified as described in Experimental Procedures. Molecular weight standards are presented.

To further support this notion, two C-terminally truncated VDAC1 constructs of about 26 kDa were designed (Figure 5A), with the aim of expressing these variants in mammalian cells, and purifying and analyzing the channel activity of the purified truncated proteins. We predicted three possible cleavage sites in membranal VDAC1, considering the estimated mass of hypoxia-induced VDAC1-ΔC, the presence of calpains both in the cytosol and in the mitochondrial intermembrane space [34] and using the Multiple Kernel Learning algorithm [47] (Figure 5A). Accordingly, we constructed two versions of VDAC1-ΔC. In the first construct, β-strands 15–19 (residues 229–283) were deleted, forming VDAC1(Δβ15–β19), while in the second construct, a shorter stretch including β-strands 16–19 (residues 241–283) was deleted to yield VDAC1(Δβ16–19). The constructs were expressed in HEK-293 cells and analyzed by immunoblotting (Figure 5B). Protein purification was initially attempted by solubilizing HEK-293 cells expressing the truncated VDAC1s with Triton X-100 or LDAO. However, these protocols were ineffective, since both VDAC1(Δβ15–β19) and VDAC1(Δβ16–β19) remained in the pellet (Figure 5C), indicating their insolubility.

**Figure 6:**
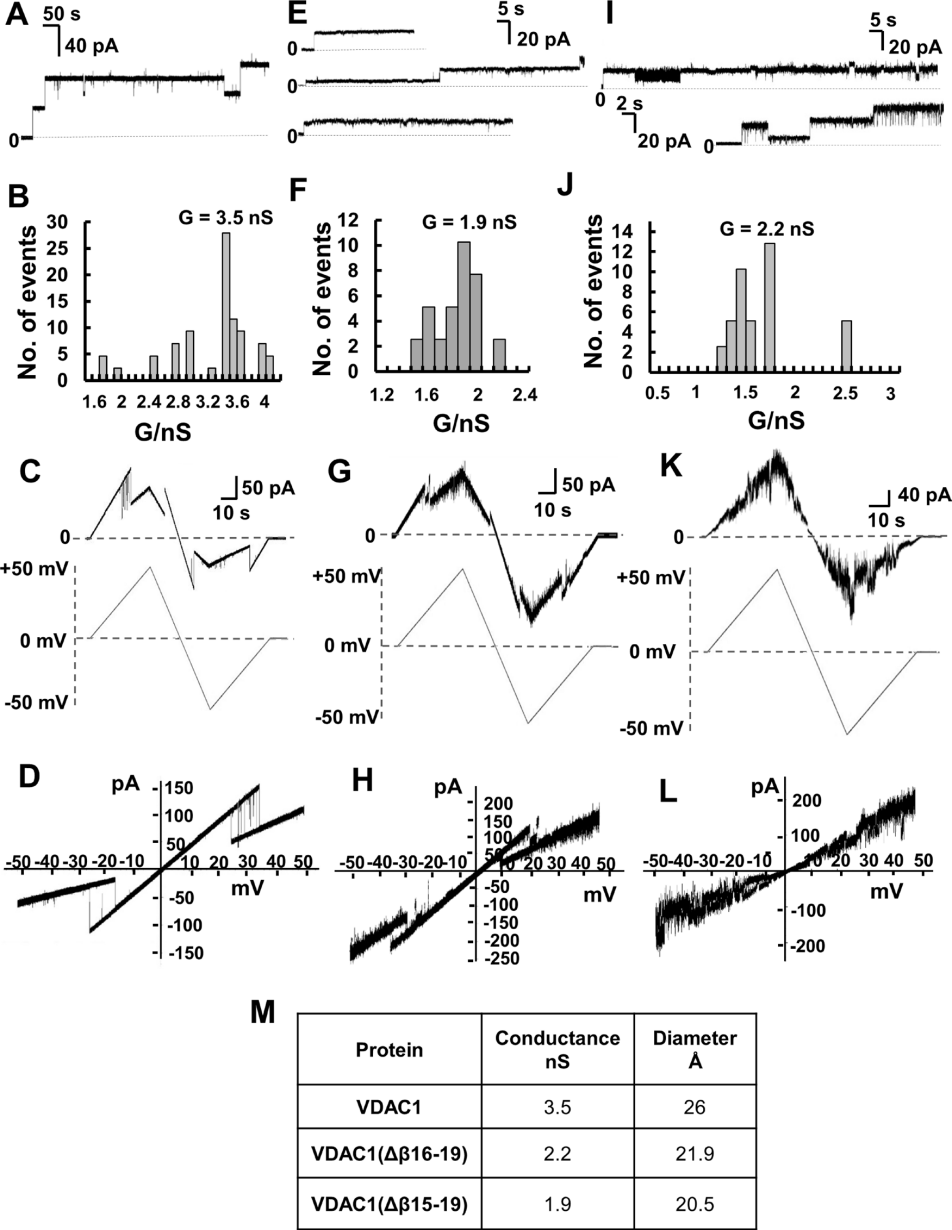
Channel properties of recombinant full-length and C-terminally truncated VDAC1 proteins Channel properties of recombinant and refolded full-length VDAC1, VDAC1(Δβ15-19) and VDAC1(Δβ16-19) reconstituted into a PLB. (**A**) Representative current traces of VDAC1 reconstituted into a DiphPC/n-decane phospholipid membrane recorded at 10 mV in 1 M KCl, 10 mM HEPES, pH 7.0, at 25° C. (**B**) Histogram of VDAC1 current amplitudes at 10 mV. The mean single channel conductance value was 3.5 nS for 40 insertion events. (**C**) Current amplitude changes across a planar bilayer containing a single VDAC1 channel in response to a linear voltage ramping between +50 mV and –50 mV. The buffer in the baths on either side of the bilayer was 1 M KCl, 10 mM HEPES, pH 7.0. (**D**) Current–voltage relationship (*I*–*V* curve) for VDAC1. (**E**) Representative current traces of VDAC1(Δβ15-19) in a PLB. Conditions as in (A). (**F**) Histogram of current amplitudes at 10 mV of VDAC1(Δβ15-19). The mean single channel conductance value was 1.9 nS for 22 insertion events. (**G**) Current amplitude changes across a planar bilayer containing a single VDAC1(Δβ15-19) channel in response to a linear voltage ramping between +50 mV and –50 mV. Conditions as in (C). (**H**) Current–voltage relationship (*I*–*V* curve) for VDAC1(Δβ15-19). (β15-19). (**I**) Representative current traces of VDAC1(Δβ16-19) in PLB. Conditions as in (A). (**J**) Histogram of VDAC1(Δβ16-19) current amplitudes at 10 mV. The mean single channel conductance value was 2.2 nS for 19 insertion events. (**K**) Current amplitude changes across a planar bilayer containing a single VDAC1(Δβ16-19) channel in response to a linear voltage ramping between +50 mV and –50 mV. Conditions as in (C). (**L**) Current–voltage relationship (*I*–*V* curve) for VDAC1(Δβ16-19). (**M**) Summary of the conductance and protein diameters of the three VDAC1 versions studied in this work. The diameters of the truncated VDAC1 versions were calculated assuming that each of them presents the same pore conformation and that each strand contributes 4.3 Å to the circumference. This figure was calculated from available VDAC1 structures, which comprise 19 β-strands, resulting in a pore circumference of 81.7 Å and a diameter of 26.0 Å (measured between Ser46 and Asn185) [63–65]. Accordingly, the calculated diameter of VDAC1(Δβ16-19] is 21.9 Å and 20.5 Å for VDAC1(Δβ15-19).

Therefore, VDAC1(Δβ15–β19) and VDAC1(Δβ16–β19) were expressed in bacteria and the recombinant proteins were purified as unfolded polypeptides that were subsequently refolded (Figure 5D). Following refolding, we analyzed the channel properties of the bacterially-expressed recombinant purified proteins following their insertion into a planar lipid bilayer (PLB) (Figure 6).

Like the full-length protein, both bilayer-reconstituted VDAC1(Δβ15–β19) and VDAC1(Δβ16–β19) were easily inserted into the artificial membranes and showed features typical of VDAC1, such as voltage-dependence conductance (Figure 6).

As with WT protein, VDAC1(Δβ15–β19) and VDAC1(Δβ16–β19) showed similar channel behavior in response to voltage stepped from a holding potential of 0 mV to +10 mV, namely, relatively stable channel with constant conductance (Figure 6A, 6E, 6I). As reported previously [48], VDAC1 conductance under the conditions used (1 M KCl, 10 mM HEPES, pH 7.0; +10 mV applied voltage) and was found to be about 3.5 nS, as expected. This value was decreased to 1.9 and 2.2 nS for VDAC1(Δβ15–β19) and VDAC1(Δβ16–β19), respectively (Figure 6B, 6F, 6J). The decreased conductance of the C-terminally truncated VDAC1 variants, relative to the conductance of native VDAC1, is in agreement with a decreased pore diameter (calculated as described in the figure legend) (Figure 6M).

Channels formed by either truncated proteins responded to changes in applied voltage, although differences between the responses of the two constructs were observed. The triangular curves obtained by constantly increasing the voltage up to +50 mV and then lowering it to -50 mV (Figure 6C, 6G, 6K) clearly showed that VDAC1(Δβ15–β19) closure was symmetric and began at ± 20 mV, while VDAC1(Δβ16–β19) exhibited high instability at every voltage applied. This is even more evident when considering the corresponding I/V graphs (Figure 6D, 6H, 6L), calculated using the data from the triangular curves. In the first case (Figure 6H), similarly to the full-length protein (Figure 6D), VDAC1(Δβ15–β19) closure corresponds to a clear change in the I/V line slope due to a reduction of current flowing through the channel. On the contrary, the absence of a net change in the VDAC1(Δβ16–β19) I/V line slope indicates that, upon increasing the applied voltage, there were no definite closure events, with the channel instead quickly switching between the closed and open states (Figure 6L). The instability of VDAC1(Δβ16–β19) did not allow us to draw definitive conclusions about its voltage dependence, however, as VDAC1(Δβ15–β19) conductance is clearly voltage-dependent, it is possible to suggest that the C-terminal β-strands are not involved in the voltage-gating mechanism. Deletion of β-strands 9–10, proposed to associate with the N-terminal moiety, has been shown to modify voltage-dependent conductance, leading to conductance asymmetry [49]. The decreased conductance of the C-terminally truncated VDAC1 variants, relative to the conductance of native VDAC1, is in agreement with a decreased pore diameter (calculated as described in the figure legend) (Figure 6M).

## DISCUSSION

A recent study showed that VDAC1-ΔC is highly expressed in advanced stage lung cancer, where tumors are known to be more hypoxic than early stage tumors, due to the large sizes of the former [19]. It was shown that the HIF-1α-dependent formation of truncated VDAC1 is associated with cell resistance to STS- and etoposide-induced cell death and truncated VDAC1 was found in patient-derived lung tumors, with truncated VDAC1 levels being increased with the disease state [19]. It is proposed that modifications in mitochondrial dynamics and production of VDAC1-ΔC are a survival response in hypoxic cancer cells that resist apoptosis [19]. Yet, the cascade of events leading to VDAC1-ΔC formation is not fully understood. Recently, a novel and compelling HIF- and TP53/TP73-dependent molecular mechanism, which implicates direct contact between mitochondria and endolysosomes in cancer cells (breast, colon, kidney, lung cancers), has been described [21, 22]. This crosstalk between organelles leads to truncation of mitochondrial VDAC1 by lysosomal peptidases and to increased resistance to chemotherapy. In this work, we focused on the effect of hypoxia applied by reduced O_2_ (1%) or as mimicked by CoCl_2_ on VDAC1 truncation and on the possible Ca^2+^ involvement in VDAC1 truncation. We have shown that hypoxia caused by either condition induced VDAC1-ΔC formation, which also resulted in the transient appearance of HIF-1α (Figure 1). As shown previously [19], the formation of VDAC1-ΔC was prevented in cells silenced for HIF-1α expression using specific siRNA (Figure 1H). Moreover, HIF-1α appearance and subsequent disappearance was shadowed by VDAC1-ΔC appearance, suggesting that the expression of HIF-1α initiates a cascade of events leading to VDAC1-ΔC formation.

We found that VDAC1 truncation requires Ca^2+^, as the Ca^2+^ chelator BAPTA-AM, decreasing [Ca^2+^]i, prevented hypoxia-induced VDAC1-ΔC formation (Figure 2A–2D). Indeed, using the genetically encoded Ca^2+^ indicator GCaMP5, an increase in the [Ca^2+^]i was visualized in cells exposed to hypoxic conditions (Figure 2F). These findings suggest that VDAC1 cleavage is mediated *via* Ca^2+^-dependent proteases. Mitochondria contain several calpains, located in both the IMS and matrix [32–34, 44]. These include μ-calpain [33, 34] and m-calpain [31, 34] located in the IMS, with the large subunit containing an N-terminal mitochondrial targeting sequence. Thus, VDAC1 can be cleaved by calpain located in the IMS or in the cytosol. However, mass spectrometry studies [21, 22] revealed that the VDAC1 cleavage site faces the cytosol.

In other studies, purified m-calpain was found to cleave isolated mitochondria-embedded VDAC1 resulting in a ~23 kDa protein band. This process required Ca^2+^ and was inhibited by PD150606, a selective non-competitive calpain inhibitor [31]. Furthermore, calpain inhibitor 1 inhibited VDAC1 truncation as activated by either CoCl_2_ or 1% O_2_ (Figure 3A–3C). Finally, using specific siRNA, we demonstrated that silencing the expression of calpain-1, but not of calpain-2, inhibited VDAC1 truncation (Figure 3D–3F), suggesting that VDAC1 can be cleaved also by calpain-1. Thus, we propose that under hypoxia, VDAC1 cleavage is mediated by both calpain-1 and AEP.

The involvement of calpain-1 in VDAC1 cleavage is not surprising due to its involvement in numerous physiological and pathological phenomena [36], including hypoxia, via participation in the destruction of HIF-1α [38], as well as its location in the IMS. Calpains were also shown to regulate cell proliferation and apoptosis through the tumor suppressor protein p53 [50]. Indeed, activation of calpains by cisplatin and oxaliplatin has been connected to apoptosis induction [51].

Based on the use of siRNA specific to the endolysosomal asparagine endopeptidase (AEP), it was recently proposed that upon hypoxia, AEP enzyme is responsible for VDAC1 truncation during local microfusion between mitochondria and endolysosomes [21, 22]. Indeed, the use of an AEP-specific inhibitor completely prevented the formation of VDAC1-ΔC (Figure 4). VDAC1 can be exposed to the AEP not only via mitochondria-endolysosome microfusion but also via the recently proposed pathway of mitochondrial-derived vesicle (MDVs)-mediated fusion with the endomembrane system [52].

We found that AEP inhibitor partially inhibited calpain activity (Figure 4C). Therefore, it is possible that some of the inhibition of VDAC1 truncation mediated by the AEP-I was a result of calpain inhibition.

Finally, hypoxia was shown to up-regulate calpain activity and mRNA expression levels in pulmonary artery endothelial cells [46]. This, together with our finding that both calpain inhibitor and down-regulation of calpain-1 expression inhibited VDAC1 truncation, lead us to propose that calpain mediates VDAC1 cleavage upon hypoxia. Thus, our findings and previously reported results [21, 22] support the involvement of both calpain and AEP in mediating VDAC1 truncation in hypoxia.

### Targeting VDAC1 to mitochondria and truncated VDAC1 channel activity properties

We previously reported that the purified, C-terminally truncated VDAC1 formed in mammalian cells upon hypoxic conditions showed identical channel conductance and interaction with hexokinase and Bcl-2 proteins as did the native protein [19]. As this finding does not fit with the expected pore size of a significantly truncated protein (Figure 6M), we decided to construct and express two VDAC1(ΔC) versions and compare their channel properties with that of the full-length protein. Using a website (http://www.calpain.org) that predicts calpain cleavage sites and using a Multiple Kernel Learning algorithm [47], putative calpain cleavage sites that would yield the desired truncated VDAC1 forms were predicted and, consequently, two alternative truncation sites were designed. To characterize channel conductance of the truncated form of the protein, the two VDAC1-ΔC versions were expressed in mammalian cells, yet were found to be insoluble (Figure 5). The truncated proteins expressed in the cells were not targeted to the mitochondria and instead were insoluble and aggregated.

Nonetheless, the bacterially-expressed proteins were soluble, and following refolding, showed voltage-dependent channel conductance upon reconstitution into a PLB (Figure 6). This is expected as the VDAC1 proteins possesses the N-terminal domain proposed to control voltage-gating [26, 53, 54]. The results also suggest that the C-terminal domain, as part of the β-barrel, serves no function in channel voltage-gating.

Channel conductance of the truncated proteins, missing β-strands 15 to 19 or 16 to 19, was only 45–63 % of the conductance of the full-length protein (Figure 6F–6L). This correlates with the decrease in pore diameter of the truncated proteins (Figure 6M). In this respect, deletion of β-strands 9 and 10 resulted in decreased channel conductance and also modified VDAC1 voltage-dependence to produce asymmetrical behaviour, proposed to be consistent with the role of these strands in stabilizing contacts with the N-terminal region of the protein [48].

Interestingly, the truncated VDAC1(Δβ15–β19), with an even number of β-strands (14), behaved more stably, with respect to channel activity, than did VDAC1(Δβ16–β19), with an odd number of β-strands (15). This observation is in agreement with the general phenomenon of even numbers of β-strands typical of bacterial porins and indicates that the exceptional case of eukaryotic VDACs may reflect evolutionarily-related functional importance. It is also of general interest that polypeptides containing repetitions of amphipathic segments of the length required to form β-strands have the potential to form membrane-inserted pores (for further discussion, see [55].

Previously, we observed canonical VDAC1 electrophysiological properties for truncated VDAC1-ΔC purified from cells subjected to hypoxia [19]. This was unexpected, as the reconstituted protein, lacking 4 to 5 β-strands, is predicted to form a channel pore smaller than that of the 19-β-strand protein (Figure 6M). Therefore, we now suggest that purified hypoxia-induced VDAC1-ΔC reconstituted into the PLB contains the two peptides (a large N-terminal peptide and a smaller C-terminal peptide). These two fragments remain associated during solubilization of the membrane-embedded protein and purification on hydroxyapatite. In SDS-PAGE, [19], the small C-terminal peptide may have eluted from the gel or was not stained. Thus, in our previous study [19], we, in fact, analyzed the channel activity of the 19-β-strand protein reconstituted into a PLB and obtained the same channel properties as the intact protein. This suggests that under hypoxic conditions VDAC1, cleaved at a single site, acts like the intact protein with respect to conductance, voltage dependence and interaction with Bcl-2.

To conclude, we propose that hypoxia leads to an increase in HIF-1α and cellular Ca^2+^ levels. This, in turn, activates a calpain1 that eventually cleaves VDAC1. In parallel, AEP also mediates VDAC1 cleavage *via* increased mitochondria-endolysosome microfusion and/or *via* fusion of mitochondrial-derived vesicles with the endomembrane system. The relationship between hypoxia and activation of the two proteolytic enzymes to cleave VDAC1 is unclear. However, the morphology of enlarged mitochondria induced by hypoxia [19] may allow an access of calpain and AEP to VDAC1, thereby its cleavage.

It was suggested that an increase in [Ca^2+^]i levels is a primary response of many cell types to hypoxia [56, 57]. We also showed that [Ca^2+^]i is increased under hypoxia (Figure 2F), and Ca^2+^ is required for VDAC1 cleavage (Figure 2A–2D), Thus, the link between hypoxia and the activation of calpain could be the increase in [Ca^2+^]i levels that, in turn, activates calpain, leading to VDAC1 cleavage. In addition, as AEP inhibitor completely eliminated VDAC1 cleavage (Figure 4A, 4B), and as AEP is not a Ca^2+^-dependent enzyme, it is possible that Ca^2+^ increases mitochondrial-endolysosomal association. To confirm this hypothesis, additional study is required.

## MATERIALS AND METHODS

### Materials

Cobalt chloride (CoCl_2_), EGTA, leupeptine, phenylmethylsulfonyl fluoride (PMSF), Triton-X100 and Suc-LYAMC calpain substrate were obtained from Sigma. N, N-Lauryl-(dimethyl)-amineoxide (LDAO) was purchased from Fluka (Buchs, Switzerland). A calpain activity assay kit (ab65308), monoclonal anti-VDAC1 antibodies directed against the N-terminal region of VDAC1 (ab154856), polyclonal anti-VDAC1 antibodies directed against the C-terminus (ab28777) and polyclonal antibodies against calpain-1 (ab28258) were purchased from Abcam (Cambridge, UK). Polyclonal antibodies against calpain (sc-30064) were purchased from Santa Cruz Biotechnology (Santa Cruz, CA), while monoclonal antibodies against HIF1-α (MAB5382) were purchased from Millipore (Billerica, MA). Horseradish peroxidase (HRP)-conjugated anti-mouse antibodies were from Promega (Madison, WI) or Abcam (Cambridge, UK). HRP-conjugated anti-rabbit antibodies were from Abcam (Cambridge, UK) or KPL (Gaithersburg, MD). Monoclonal anti-actin antibodies (MAB1501) were from Millipore. Dulbecco's modified Eagle's medium (DMEM) was purchased from Gibco (Grand Island, NY), while normal goat serum (NGS), fetal calf serum, L-glutamine, and penicillin-streptomycin solution were purchased from Biological Industries (Beit Haemek, Israel). Blasticidin was purchased from InvivoGen (San Diego, CA). Calpain-1- and -2-specific siRNA were synthesized by Genepharma (Suzhou, China) and HIF-1α–specific siRNA was obtained from (IDT). Jet-Prime transfection reagent was obtained from Polyplus. Transfection (Illkirch, France). BAPTA-AM (1,2,-Bis(2-aminophenoxy) ethane-N,N,N',N'-tetra acetic acid tetrakis (acetoxymethyl ester)) was from Tocris Bioscience (Bristol, UK) and N-Acetyl-Leu-Leu-norleucinal (calpain inhibitor 1) was obtained from Boehringer Ingelheim (Ingelheim am Rhein, Germany). Asparagine endopeptidase inhibitor was kindly provided by Prof. Colin Watts, University of Dundee, Dundee, UK. pGCaMP5 was a gift from Sebastian Lourido (Addgene plasmid # 78705).

### Cell lines and hypoxia

HeLa and HEK-293 cells were grown in DMEM supplemented with 10% fetal calf serum, 100 U/ml penicillin and 100 μg/ml streptomycin, and maintained in a humidified atmosphere at 37° C with 5% CO_2_. Hypoxic conditions were achieved by incubating the cells in 1% O_2_, 5% CO_2_, 94% N_2_, using a Nuaire incubator (NU-5841A). HeLa cells were incubated for 48 h under hypoxic conditions in DMEM medium containing 12 mM glucose and supplemented with 10% fetal calf serum, 100 U/ml penicillin and 100 μg/ml streptomycin. Hypoxic condition mimicry was achieved using CoCl_2_ [39]. HeLa cells were incubated overnight (16 h) with DMEM containing 100 U/ml penicillin, and 100 μg/ml streptomycin (without 10% FCS), and then CoCl_2_ solution was added to different final concentrations (50–200 μM), followed by incubation for various periods, as indicated in the tables and figure legends.

### Calpain and HIF1-α silencing by siRNA transfection

To silence calpain expression, the following human (h)Calpain-1-siRNAs (si-hCalpain-1) and hCalpain-2-siRNA (si-hCalpain-2) sequences were used: hCalpain1- sense: 5′-AAGCUAGUGUUCGUGCACUC U-3′, anti-sense:

5′-AGAGUGCACGAACACUAGCUU-3′ and hCalpain-2 - sense: 5′-AAACCAGAGCUUCCAG GAAA A-3′ and anti-sense: 5′-UUUUCCUGGAAGCUCUGG UUU-3′.

To silence HIF1-, α sense: 5′-ACAGAUUUAG ACUUGGAGAUGtt -3′ and anti-sense: 5′ CAUCUCCA AGUCUAAAUC UGUtt-3′ sequences were used. Cells were seeded (120,000–150,000 cells/well) in 6-well culture dishes to 40–60% confluence and transfected with 50 or 100 nM siRNA against si-hCalpain1, si-hCalpain-2 or HIF1-α using the JetPRIME transfection reagent, according to the manufacturer's instructions.

### Measurement of calpain activity

Cells (7 × 10^6^/ml) were rinsed, scraped, and homogenized in extraction buffer (10 mM Tris-HCl, pH 7.5). The homogenate was then centrifuged at 14,000 rpm for 10 min, and 50 to 100 μl of the supernatant, or purified calpain was assayed for activity. The reaction mixture included (final concentrations) 200 mM Tris-HCl, pH 8.0, 20 mM CaCl_2_, 10 mM cysteine and 50 μM Suc-LYAMC (Sigma) as substrate. Activity was monitored as the change in fluorescence as a function of time measured at excitation/emission wavelengths of 345 nm and 445, respectively, in a 96-well plate using a Synergy microplate reader. Purified calpain and its activity was measured using a calpain activity assay kit (ab65308) as described in the manufacturer's instructions.

### Monitoring changes in intracellular calcium levels using GCaMP5

HeLa cells were transfected with plasmid p-GCaMP5 (1 μg) using the JetPRIME transfection agent and were grown in normoxic conditions. Forty-eight h post-transfection, the cells were transferred to hypoxic condition (1% O_2_) for 12, 24 or 48 h. For imaging, cells were removed from the incubator and immediately examined with an IX51 fluorescence microscope (Olympus) equipped with a CCD camera, using an excitation of 475 nm and emission of 525 nm.

### VDAC1(Δβ15-19), VDAC1(Δβ16-19) construction and cell expression

To generate two versions of truncated rat (r)VDAC1, the sequences of interest were amplified by PCR from rat (r)VDAC1 cDNA (Table 1), sub-cloned into the *Bam*HI*/Not*I sites of the tetracycline-inducible pcDNA4/TO vector, and cloned into the *Bam*HI*/Not*I sites of the pcDNA3.1 and the *Xho*I/*Bam*HI sites of the pEGFP-N1 plasmid.

**Table 1:** Primers used for VDAC1-ΔC construction

Primer	Sequence
VDAC1(ΔC) BamH1 F	AAAGGATCCATGGCTGTGCCACCCACGTATG
VDAC1(ΔC) Not1 R (15)	TTTGCGGCCGCTTAAGGGTCGACCTGATACTTGGCTG
VDAC1(ΔC) Not1 R (16)	TTTGCGGCCGCTTAACTGGAGTTGTTCACTTTGGCC
VDAC1(ΔC) F Xho1	CATCTCGAGATGGCTGTGCCTCCCACATATG
VDAC(Δ15-19) R BamH1	CATGGATCCGCAGGGTCGACCTGATACTTGG
VDAC(Δ16-19) R BamH2	CATGGATCCGCGGAGTTGTTCACTTTGGCCG

HEK-293 cells were transfected with empty pEGFP-N1 plasmid as a control or with pcDNA3.1 plasmids encoding for native or truncated rVDAC1. Transfection solution contained 0.3–1 mg plasmid in 25 mM Hepes pH 7.05), 120 mM CaCl_2_, 140 mM NaCl, 5 mM KCl, 0.75 mM Na_2_HPO_4_, and 6 mM glucose. The transfection solution was added to each culture plate (~6 × 10^5^ cell per well in 6-well plates) containing 2 ml of fresh DMEM medium supplemented with 10% FCS, 1 mM L-glutamine, 100 U/ml (1%) penicillin, and 100 mg/ml (1%) streptomycin. After 16 h of incubation, 2 ml of fresh DMEM medium supplemented with 10% FCS, 1 mM L-glutamine, 100 U/ml (1%) penicillin, and 100 mg/ml (1%) streptomycin were added. To detect expression levels of VDAC1 or truncated rVDAC1, cells were lysed and the resulting lysates were analyzed by SDS-PAGE and immunoblotting.

### Cloning, heterologous expression and purification of full-length VDAC1 and of the C-terminally truncated proteins VDAC1(Δβ15-19) and VDAC1(Δβ16-19)

cDNAs encoding full-length VDAC1 and the C-terminally truncated proteins VDAC1(Δβ15-19) and VDAC1(Δβ16-19) were cloned into the pET21a vector (Novagen) in-frame with DNA encoding a C-terminal 6xHis-tag, using the *Nhe*I/*Xho*I sites. All constructs were verified by sequencing. Refolded VDAC isoforms were obtained essentially as described [58]. In brief, *Escherichia coli* BL21(DE3) cells were transformed with plasmid pET21a containing VDAC isoform-coding sequences. Protein expression was induced for 3 h by addition of 1 mM isopropyl-β-D-thiogalactopyranoside (IPTG, Sigma) when the cultures reached an optical density (λ = 595 nm) of ~0.6 at 37° C, as reported [59]. The cells were resuspended in 8 M urea in buffer B (100 mM Tris /HCl, 10 mM NaH_2_PO_4_, pH 8.0), and shaken overnight at 4° C. After pelleting cell debris by centrifugation, the clear lysate was loaded onto a 1 ml of agarose-packed nickel-nitrilotriacetic acid resin (Ni-NTA; Qiagen) in a glass 2.5 × 30 cm Econo-column (BioRad), pre-equilibrated with 20 volumes of buffer B. The column was then washed with 10 volumes of buffer C (8 M urea, 10 mM NaH_2_PO_4_, 100 mM Tris-HCl, pH 6.2) and the captured proteins were eluted with 5 volumes of the same solution at pH 3.5. Fractions containing purified His-tagged proteins were analyzed using SDS-PAGE (4–12% acrylamide) and Coomassie Blue staining.

Refolding of purified proteins was carried out as described [60]. The denatured protein mixture was added drop-wise to refolding buffer (25 mM Tris-HCl, pH 7.0, 100 mM NaCl, 1 mM EDTA, 1% (v/v) LDAO) to obtain a ten-fold dilution of the urea concentration, and gently stirred overnight at 4° C. The protein solution was dialyzed against 100 volumes of a dialysis buffer (25 mM Tris-HCl, pH 7.0, 1 mM EDTA, 0.1% LDAO) in Thermo Scientific Slide-A-Lyzer Dialysis Cassettes (3.5 kDa MWCO), changing the dialysis buffer two times after 2 h of stirring at 4° C and once more, 24 h later. Protein purity was verified by SDS-PAGE and Coomassie staining. Purified samples were stored at –20° C.

### Cell lysate preparation

Following the desired treatment, cells were harvested using trypsin, centrifuged for 5 min at 1500g, and washed twice with PBS. Cells were then resuspended with lysis buffer containing 20 mM HEPES, pH 7.4, 150 mM NaCl, 1 mM EDTA, 1 mM EGTA, 10% glycerol, and 1 mM MgCl_2_. Samples were then sonicated for 10 sec, centrifuged for 10 min at 600g, and the supernatant (lysate) was collected. Protein concentration in the sample was determined using the Bradford method [61] and samples were stored at –20° C until use. For electrophoresis, samples were diluted 4:1 with 4× sample buffer containing 40% glycerol, 4% β-mercaptoethanol, 8% SDS, 0.26 M Tris-HCl, pH, 6.8 and bromophenol blue, and incubated for 10 min at 70° C.

### Gel electrophoresis and immunoblot analysis

SDS-PAGE was performed according to the Laemmli protocol [62]. For immunostaining, membranes containing electro-transferred proteins were incubated with a blocking solution containing 5% non-fat dry milk and 0.1% Tween-20 in Tris-buffered saline, followed by incubation with monoclonal anti-VDAC1 (1:4,000), anti-actin (1:40,000), anti-calpain 1 (1:4000), anti-calpain (1:1000) or anti-HIF 1-α (1:2000) antibodies. Membranes were then incubated with HRP-conjugated anti-mouse/rabbit IgG (1:15,000) as a secondary antibody. Antibody binding was detected using an enhanced chemiluminescent assay (Pierce Rockford, IL) for detection of HRP. Imaging and quantitative analysis were obtained using FUSION-FX (Vilber Lourmat, France).

### VDAC channel reconstitution, recording and analysis

Electrophysiological properties of purified refolded full-length VDAC1 and of the C-terminally truncated proteins VDAC1(Δβ15-19) and VDAC1(Δβ16-19) reconstituted into a PLB and subsequently subjected to single and multiple channel current recordings and data analysis were carried out as described previously [58, 60], using a Warner Instruments (Hamden, CT) planar bilayer apparatus. Bilayers of approximately 150–200 pF capacity were prepared across a 200 μM hole in a derlin cuvette (Warner Instruments) from a 1% (w/v) solution of DiPhyPC (1,2-diphytanoyl-sn-glycero-3-phosphocoline) (Avanti Polar-Lipids, Alabaster, AL) in n-decane (Sigma). The volumes of the *cis* and *trans* compartments were 3 ml. Both sides were connected to the electrodes via salt bridges (1 M KCl) in series with Ag/AgCl electrodes. All measurements were made in 1 M KCl, 10 mM Hepes, pH 7.0, at room temperature. Full-length and truncated VDAC were added to the *cis* side of the chamber from a protein stock solution of 1 mg/ml. Control experiments with a PLB in the absence of VDAC1 or in the presence of the detergent used for VDAC1 purification showed no currents. Data were acquired using a Bilayer Clamp amplifier (Warner Instruments) at the 100 μs/point, filtered at 200 Hz and analyzed offline using pCLAMP Clampfit 10.7 software (Axon Instruments, Union City, CA).

### Statistical analysis

Data are expressed as means ± SE. Statistical evaluation was carried out using Student's *t* test (two-tailed) to test for differences between control and experimental results. A difference was considered statistically significant when the *p* value was ^*^P ≤ .0.01, ^**^P ≤ .001; ^***^P ≤ .0001.

## Abbreviations

AEP: asparagine endopeptidase
VDAC1: voltage-dependent anion channel 1
